# Linkage disequilibrium and diversity for three genomic regions in Azoreans and mainland Portuguese

**DOI:** 10.1590/S1415-47572009000200003

**Published:** 2009-06-01

**Authors:** Claudia C. Branco, Paula R. Pacheco, Ester Cabrol, Rita Cabral, Astrid M. Vicente, Luisa Mota-Vieira

**Affiliations:** 1 Molecular Genetics and Pathology Unit, Hospital of Divino Espírito Santo of Ponta Delgada, São Miguel Island, AzoresPortugal; 2 Instituto Gulbenkian de Ciência, OeirasPortugal; 3 Instituto Nacional de Saúde Dr. Ricardo Jorge, LisboaPortugal

**Keywords:** linkage disequilibrium, X-chromosome, Y-chromosome, HLA, São Miguel, Azores

## Abstract

Studies on linkage disequilibrium (LD) across the genome and populations have been used in recent years with the main objective of improving gene mapping of complex traits. Here, we characterize the patterns of genetic diversity of HLA *loci* and evaluate LD (D') extent in three genomic regions: Xq13.3, NRY and HLA. In addition, we examine the distribution of DXS1225-DXS8082 haplotype diversity in Azoreans and mainland Portuguese. Allele distribution has demonstrated that the São Miguel population is genetically very diverse; haplotype analysis revealed 100% discriminatory power for X- and Y-markers and 94.3% for HLA markers. Standardized multiallelic D' in these three genomic regions shows values lower than 0.33, thereby suggesting there is no extensive LD in the São Miguel population. Data regarding the distribution of DXS1225-DXS8082 haplotypes indicate that there are no significant differences among all the populations studied, (Azorean geographical groups, the Azores archipelago and mainland Portugal). Moreover, in these as well as in other European populations, the most frequent DXS1225-DXS8082 haplotype is 210-219. Even though São Miguel islanders and Azoreans do not constitute isolated populations and show LD for only very short physical distances, certain characteristics, such as the absence of genetic structure, the same environment and the possibility of constructing extensive pedigrees through church and civil records, offer an opportunity for dissecting the genetic background of complex diseases in these populations.

## Introduction

Linkage disequilibrium (LD), the nonrandom association of alleles at different *loci*, varies across populations and genomic regions, as well as between pairs of markers in close proximity. Certain factors which generate LD variance, as for example genetic drift and admixture, are population specific. Others, such as recombination rate, gene conversion and natural selection, are specific to genomic regions (Shifman *et al.*, 2003). For these reasons, studies on LD extent and population structure are a good starting point for the investigation of complex traits (Angius *et al.*, 2002).

The Azores is a Portuguese archipelago composed of nine islands distributed among three geographical groups: the Eastern group with two islands, São Miguel and Santa Maria; the Central group which includes five islands, Terceira, Pico, Faial, São Jorge and Graciosa; and the Western group, with Flores and Corvo islands. The Portuguese explorers, who discovered the archipelago in 1427, only started the settlement in 1439 through a long and difficult process. Historical data report that the Portuguese crown was compelled to cede both land and privileges, not only to the Portuguese, but also to foreigners, so as to attract people to the islands. Thus the Azorean population received a significant contribution from people with genetic backgrounds other than Portuguese. This included individuals of Flemish, Spanish, French, Italian, German, Scottish and Jewish origins, as well as Moorish prisoners and black slaves from Guinea, Cape Verde and São Tomé (Guill, 1993). The first islands to be settled were Santa Maria and São Miguel, the last being Flores and Corvo in the beginning of the 16^th^ century, the latter being populated mainly with individuals from the other islands.

São Miguel is the largest island of the Azores, with a population of 131,609 inhabitants (2001 Census, Portugal National Institute of Statistics) derived from around 27 generations. Several studies (Santos *et al.*, 2003; Montiel *et al.*, 2005; Pacheco *et al.*, 2005; Spínola *et al.*, 2005a; Branco *et al.*, 2006, 2008a, 2008b, 2008c) have been undertaken, with the aim of characterizing the genetic pool of the Azoreans. These studies report high genetic variability and heterogeneity in the Azorean population, as explained by the history of the settlement of the islands. Recently, Laan *et al.* (2005) proposed that the evaluation of DXS1225-DXS8082 haplotype diversity constitutes an efficient marker of population genetic history due to its low recombination rate. Therefore, in order to unravel possible differences between mainland Portuguese and Azoreans, unobserved in previous works, we re-analysed the data published in Branco *et al.* (2008a) for the three Azorean geographical groups, as well as for the Azores archipelago and mainland Portugal. In addition, the present research, based on an analysis of the Xq13.3 non-recombining portion of the Y-chromosome (NRY) and HLA regions in São Miguel Islanders, also mainly aims at answering questions such as: What is the allelic distribution of HLA class I and II in this island population, and does it reveal the presence of a genetic structure? Does LD extent vary considerably between these three different genomic regions?

**Figure 1:**
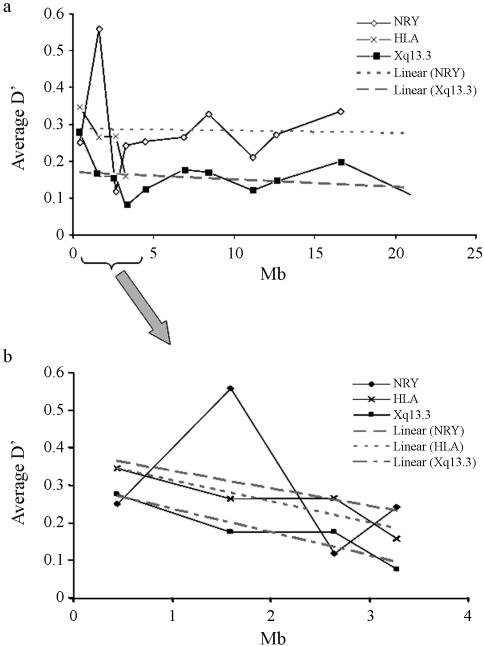
Comparison of the LD extent in Xq13.3, NRY and HLA regions, evaluated as average multiallelic D' values *vs.* physical distances for the São Miguel Island population. Figure 1b is an expanded view of the 1-5 Mb section of 1a. There is a decrease in LD values for shorter distances (< 4 Mb) in all markers.

## Material and Methods

### Population samples and genotyping

The sample set was composed of healthy blood donors living in São Miguel Island and obtained from the “anonymous” Azorean DNA bank located in the main Hospital of the Azores archipelago, Portugal (Mota-Vieira *et al.*, 2005). LD for X- and Y-chromosomes was assessed only in males (189 and 149, respectively), whereas the analysis of the HLA region consisted of 106 individuals of both sexes (8 females and 98 males). The Xq13.3 region was analyzed according to Branco *et al.* (2008a), by examining eight microsatellite markers - DXS983, DXS1066, DXS986, DXS8092, DXS8082, DXS1225, DXS8037 and DXS995 - spanning approximately 6.9 centiMorgans (cM) or 20.9 megabases (Mb). Moreover, DXS1225-DXS8082 haplotype frequencies were estimated in the three Azorean geographical groups (Western, Central and Eastern), as well as in the Azores archipelago and mainland Portugal, by using a total of 527 individuals (450 islanders and 97 mainlanders), the same as were previously studied by these authors (Branco *et al.*, 2008a).

The characterization of 7 Y-STRs in 172 male individuals is described in Pacheco *et al.* (2005). HLA class I (-A, -B and -Cw) and class II (-DRB1, -DQB1, -DPA1 and -DPB1) genotyping was undertaken in 106 individuals by PCR-SSP Olerup SSP (GenoVision Inc.), according to manufacturers' instructions. After electrophoresis on a 4% agarose gel stained with SYBR® Green, the PCR products were visualized, followed by HLA allele identification using the Helmberg-SCORE “Sequence Compilation and Rearrangment Evaluation for Research only” software version 3.320T (Olerup SSP AB, Saltsjöbaden, Sweden). We also typed two dinucleotide STRs - D6S265 and TNFα - located in the HLA region Branco *et al.* (2008b).

### Statistical analysis

Allele and DXS1225-DXS8082 haplotype frequencies were calculated by direct counting. Average gene diversity estimation was done by using Arlequin software. Estimation of HLA haplotypes was obtained through the expectation maximization (EM) algorithm, an iterative procedure from multilocus genotype data, with the unknown gamete phase implemented in Arlequin v3.0. Evaluation of standardized multiallelic disequilibrium coefficient, D', was performed using the Haploxt application from GOLD software. This program calculates disequilibrium statistics from haplotype data. An estimation of average D' values in each genomic region, was executed with a simple mathematical mean, for all values obtained for each marker pair. Since the extent of the three regions varied widely, a second analysis, taking into consideration a smaller genetic distance of around 3 Mb, was undertaken, so as to give further insight into LD patterns over short distances, and to guarantee the truthfulness of the drawn conclusions.

## Results

### HLA diversity in São Miguel Island

An analysis of the HLA alleles in 106 individuals from São Miguel Island (Table 1) revealed that the average gene diversity for all HLA *loci* varied from 0.821 for -DQB1 and -DPA1 to 0.934 for -B. Considering HLA *loci,* the overall gene diversity for São Miguel islanders was 0.843 (Table 2). HLA allele frequencies in São Miguel, mainland Portugal and other European populations demonstrated the absence of statistically significant differences (G_ST_ = 0.03; data not shown).

Haplotype analysis reveals a total of 200 HLA haplotypes, corresponding to 94.3% discriminatory power. Only the three most representative HLA-A-B-DRB1 haplotypes where evaluated (data not shown and provided as supplementary information by the authors upon request). A*01-B*08-DRB1*03 is the most frequent haplotype in São Miguel (8%) followed by A*02-B*44-DRB1*04 and A*02-B*44-DRB1*07, both with 1.4% frequency. Comparison with other European and African populations (Arnaiz-Villena *et al.*, 1995, 1997, Martinez-Laso *et al.*, 1995) revealed 3 haplotypes, unique to the São Miguel Island population, namely A*02-B*44-DRB1*07, A*02-B*44-DRB1*11 and A*02-B*44-DRB1*15.

### Linkage disequilibrium in São Miguel Island

Since LD varies among genomic regions within the same population, in São Miguel Island, we investigated the extent of this parameter in the Xq13.3, NRY and HLA regions. The number of haplotypes, genetic diversity and D' average values are shown in Table 2. Taken as a whole, diversity results demonstrate that the São Miguel Island population is very diverse. In terms of haplotype number, which in itself can influence the value of D', we observed a smaller value for the NRY region. Since LD is generated by evolutionary processes, it is important to assess the patterns of LD, both in sex and autosomal chromosomes. In a comparison of D' in Xq13.3, both the NRY and HLA regions reveal lower LD than the Xq13.3 (Table 2). The data indicate a higher LD for the NRY, followed by the HLA region.

On examining the analysis of LD patterns in shorter genetic distances (~3 Mb), we observed the same trend. Nevertheless, values had increased for both Xq13.3 and NRY. The value for HLA had diminished due to the cut-off genetic distance value being smaller than that evaluated previously. The average D'_2_ (0.247; Table 2) was not statistically different when compared to the observed value in larger genetic distance analysis (0.243; ~21 Mb).

Figure 1a shows plotting average D' over physical distances. We detected a decrease in LD values for shorter distances (< 4 Mb) in all the regions. As expected, the highest value (> 0.5) obtained in the X-chromosome corresponds to the association DXS1225-DXS8082, which is the shortest physical distance between all the markers. In order to compare LD values, when taking into consideration the shortest distance studied (~3 Mb), we analysed D' for the three genomic regions (Figure 1b). The results indicated the same tendency. We also added trend lines (grey lines; Figures 1a and 1b), so as to understand the distribution of D' values in these genomic regions. The results clearly demonstrated that D' values diminished with physical distance. Nevertheless, we did not consider a trend line for HLA *loci*, in Figure 1a, since it would present a sharp decline, tending to negative values. We also noticed that, in both the NRY and Xq13.3 regions, stabilization in D' values with physical distance was registered (grey lines; Figures 1a and 1b), probably due to less recombination in the Xq13.3 region and a lack in the NRY.

### DXS1225-DXS8082 haplotype analysis in Azorean and mainland Portuguese populations

Several studies (Latini *et al.*, 2004; Bellis *et al.*, 2008) have demonstrated a firm association between the markers DXS1225-DXS8082 (Xq13.3), mainly as a result of their short physical distance (162 kb). Recently, Laan *et al.* (2005) proposed this haplotype as a good marker of population genetic history, due to its low recombination rate. The distribution of DXS1225-DXS8082 haplotypes was also analyzed (Table 3) in Azoreans and mainland Portuguese populations, in order to detect significant differences between islanders and mainlanders. The results demonstrated that the most frequent haplotype in the Azorean and mainland Portuguese populations was 210-219 followed by 192-229. We identified a total of 52 different DXS1225-DXS8082 haplotypes, but only 16 with a frequency of ≥3% (Table 3). Based on sample distribution, this selection of criterion (≥3%) allows us to illustrate certain differences between the Azorean and mainland populations. For instance, we could only observe the presence of haplotypes 192-219 and 214-219 in the Western, 212-219 in the Central, and 198-225 in the Eastern groups. From Table 3, we could also perceive that there are only 3 common haplotypes in the whole Azorean population and geographical groups, namely 210-219 (the most frequent), 192-229 (the second most frequent) and 192-231 (absent in mainland Portugal). In the Azores archipelago population, we observed the presence of 9 out of 16 different haplotypes. If we add those with a frequency of ≥1% (data not shown), we could identify 17 out of 52 (the remaining 35 do not reach 1% frequency). On considering the ≥3% criterion, mainland Portugal shows 8 out of 27 different haplotypes (Table 3), all with a frequency of ≥1% (data not shown).

## Discussion

The evolution of populations is dependent on several mechanisms, such as migration, genetic drift, selection and mutation, all affecting the patterns of diversity of neutral and disease variants. Consequently, the measure of diversity in neutral markers allows for inferring how these processes shape the overall signature of a population, besides deducing further implications in general disease apportionment, since non-neutral *loci* may be under the same evolutionary forces. In general, the data corroborate previous studies (Pacheco *et al.*, 2005; Branco *et al.*, 2006; Branco *et al.*, 2008a, 2008b, 2008c), where Azoreans and São Miguel islanders showed higher genetic diversity values than mainland Portugal and other European populations. This may be a direct consequence of the Azores settlement process, where a major contribution of mainland Portuguese and, to a lesser extent, Flemish, Spaniards, French, Italians, Germans, Scots, Jews, Moors and Negroes from Guinea, Cabo Verde and São Tomé is reported. Previous studies of HLA markers in mainland Portugal (3 *loci,* -A, -B and -DRB1; Spínola *et al.*, 2005b) and in Azores (6 *loci,* -A, -B, -Cw, -DRB1, -DQA1 and -DQB1; Spínola *et al.*, 2005a) demonstrate average diversity values of 0.92 in the two populations. The results obtained in the present study, based on 7 *loci*, showed a lower value (0.84). This may be explained by the difference in number of analysed *loci* and by the fact that Spinola *et al.* (2005a) used a high-resolution methodology for the HLA genotype. In addition, the same authors (Spínola *et al.*, 2005a) question the identification of paternal lineage N3, specific to Asians and north Europeans (Helgason *et al.*, 2000; Rosser *et al.* 2000), since, based on HLA *loci*, they did not encounter any results supporting this observation. Historic records reporting the presence of Asians or Mongolians in the archipelago are unknown. Nevertheless, with HLA data, the haplotype A*02 B*44 DRB1*04 was identified at a frequency of 1.42%. This haplotype, possibly oriental in origin, has previously been described in the Azores (Bruges-Armas *et al.*, 1999). The introduction of this genetic contribution probably occurred during the expansion of trade navigation between Europe, America and Asia, in the 16^th^ and 17^th^ centuries, when the Azores played a strategic role due to its geographic position.

Meyer *et al.* (2006) investigated LD among all the HLA *loci* in around 40 populations worldwide, and reported significant LD values. The present results, although showing that HLA has significant pairwise LD *p*-values (p < 0.01; 13 out of 36 pairs with significant LD; data not shown), do not imply strong LD for this region (D' values < 0.3). The distribution of LD among Y-linked alleles is accepted as being substantially larger than that of X-linked markers, since, Y-alleles constitute/have only one-fourth of the effective population size. This assumption is confirmed by data obtained herein. Nevertheless, the highest peak identified in Figures 1a and 1b corresponds to the association between DYS392-DYS385. This was unexpected, since this region does not present recombination. We hypothesise that this observation may reflect the influence of stochastic processes, such as random sampling, or even point mutations throughout the evolution of populations, since these markers have a higher genetic distance (~1.75 Mb) when compared to markers with the lowest value (DYS389I-DYS389II; ~0.25 kb).

The study of DXS1225-DXS8082 haplotype diversity in Azorean and mainland Portuguese populations, has contributed to understanding how these populations are mutually related. The X-chromosome is an important tool for historical research, since there is but one copy in males, thus facilitating the determination of haplotypes. This feature permits accurately determining LD extension, and also allows for inferring population “maternal lineages“. It is clear that, since this chromosome undergoes recombination, direct maternal lineages may not be obtained. Nonetheless, on studying DXS1225-DXS8082, which has a very small probability of recombination (their physical distance is 162 kb), we could come to some interesting conclusions. Taking into account the frequency ≥1% proposed by Laan *et al.* (2005), it was possible to notice that most haplotypes were present in all the evaluated populations, thereby suggesting that there were no mutual differences. Moreover, the 210-219 haplotype, reported by Laan *et al.* (2005) as being the most widely represented in Europe, was also the most frequent in this study. These results confirm previous works, where a strong similarity between Azoreans and other Europeans was evident (Pacheco *et al.*, 2005; Branco *et al.*, 2006 2008a, 2008b, 2008c).

There is some controversy regarding the amount of useful LD for mapping studies. According to Abecasis *et al.* (2001), the value of D' = 0.33, which corresponds to a 10-fold increase in the required sample size, is commonly taken as the minimum usable amount of LD. On the other hand, Reich *et al.* (2001) consider that D' > 0.5 is useful. Although both of these D' values are estimates based on SNP (single nucleotide polymorphism) markers, Schulze *et al.* (2002), on comparing both SNPs and microsatellites, reported the same values for D'. In the present case, none of the samples studied manifested values higher than 0.5 or 0.33, thus indicating no LD for the São Miguel population. These results are corroborated by those obtained by Service *et al.* (2006) and Branco *et al.* (2008a), where the Azoreans presented the lowest values of LD when compared with isolated and outbred populations. Even though the Azores are not an isolated population, there are certain characteristics that offer the opportunity for dissecting the genetic background of complex diseases in these populations, such as the absence of genetic structure, the same environment and the possibility of constructing extensive pedigrees through church and civil records. The absence of structure reduces the presence of false genetic associations in complex disease studies. The same environment allows for better control over external factors that may be influencing the development of a complex disease. Finally, extensive pedigrees permit the development of reliable studies on linkage, with statistical significance. In summary, the overall data implies that the identification of identical-by-descent (IBD) regions surrounding disease susceptibility genes or other complex trait *loci* in the São Miguel population, as well as in Azoreans, will require very high marker density, where data from the HapMap project (The International HapMap Consortium, 2007) will most certainly increase the power of IBD mapping.

## Figures and Tables

**Table 1:** HLA class I and II allele frequencies and average gene diversity (AGD) in the São Miguel population (the highest values are in bold type).

HLA class I (2n = 212)						HLA class II (2n = 212)				
Alleles	Rel. freq.		Alleles	Rel. freq.		Alleles	Rel. freq.		Alleles	Rel. freq.
**HLA-A**			**HLA-B**			**HLA-DPB1**			**HLA- DRB1**	
A*01	0.151		B*07	0.066		DPB1*0101	0.057		DRB1*01	0.085
A*02	**0.250**		B*08	0.137		DPB1*0201	0.212		DRB1*03	0.165
A*03	0.094		B*13	0.005		DPB1*0202	0.014		DRB1*04	0.123
A*11	0.042		B*14	0.071		DPB1*0301	0.080		DRB1*07	**0.170**
A*23	0.019		B*15	0.052		DPB1*0401	**0.316**		DRB1*08	0.028
A*24	0.137		B*18	0.052		DPB1*0402	0.094		DRB1*09	0.019
A*25	0.005		B*27	0.042		DPB1*0501	0.014		DRB1*10	0.019
A*26	0.009		B*35	0.061		DPB1*0601	0.005		DRB1*11	0.118
A*29	0.066		B*37	0.014		DPB1*0901	0.005		DRB1*12	0.009
A*30	0.033		B*38	0.014		DPB1*1001	0.028		DRB1*13	0.146
A*31	0.024		B*39	0.009		DPB1*1101	0.024		DRB1*14	0.019
A*32	0.061		B*40	0.028		DPB1*1301	0.052		DRB1*15	0.075
A*33	0.028		B*41	0.024		DPB1*1401	0.014		DRB1*16	0.024
A*66	0.005		B*44	**0.156**		DPB1*1501	0.005		**AGD = 0.877**	
A*68	0.071		B*45	0.009		DPB1*1601	0.005		**HLA- DQB1**	
A*80	0.005		B*47	0.005		DPB1*1701	0.038		DQB1*02	0.302
**AGD = 0.877**			B*49	0.052		DPB1*1901	0.014		DQB1*03	**0.321**
**HLA-Cw**			B*50	0.033		DPB1*2501	0.005		DQB1*04	0.028
Cw*01	0.024		B*51	0.066		DPB1*3901	0.005		DQB1*05	0.151
Cw*02	0.066		B*53	0.024		DPB1*5101	0.005		DQB1*06	0.198
Cw*03	0.075		B*55	0.019		DPB1*6601	0.005		**AGD = 0.821**	
Cw*04	0.104		B*57	0.042		DPB1*7801	0.005			
Cw*05	0.071		B*58	0.014		**AGD = 0.906**				
Cw*06	0.090		B*78	0.005		**HLA- DPA1**				
Cw*07	**0.311**		**AGD = 0.934**			DPA1*01	**0.462**			
Cw*08	0.052					DPA1*0103	0.255			
Cw*12	0.047					DPA1*0105	0.005			
Cw*14	0.019					DPA1*0201	0.226			
Cw*15	0.047					DPA1*0202	0.042			
Cw*16	0.071					DPA1*0301	0.009			
Cw*17	0.024					**AGD = 0.821**				
**AGD = 0.839**										

**Table 2:** Number of haplotypes (NH), gene diversity (GD) and standardized multi-allelic disequilibrium coefficient (D') for three genomic regions in the São Miguel Island population.

Genomic region	NH	GD	D'_1_	D'_2_
Xq13.3	189	0.691	0.172	0.177
NRY	149	0.574	0.282	0.370
HLA	200	0.843	0.275	0.258
Average	179	0.703	0.243	0.247

D'_1_ = LD over ~21 Mb; D'_2_ = LD over ~3 Mb.

**Table 3:** Common haplotypes of micro-satellite *loci* DXS1225-DXS8082 with frequency ≥3% for Azoreans and mainland Portuguese populations.

DXS1225- DXS8082 haplotypes		Azorean geographical groups									Azores archipelago			Mainland Portugal	
		Western (N = 93)			Central (N = 150)			Eastern (N = 207)			(N = 450)			(N = 97)	
		Abs. freq.	%		Abs. freq.	%		Abs. freq.	%		Abs. freq.	%		Abs. freq.	%
192-219		3	3.23		-	-		-	-		-	-		-	-
-227		4	4.30		6	4.00		-	-		-	-		3	3.09
-229		4	4.30		10	6.67		21	10.14		35	7.80		16	16.49
-231		4	4.30		7	4.67		13	6.28		24	5.35		-	-
198-221		-	-		-	-		13	6.28		16	3.56		3	3.09
-225		-	-		-	-		8	3.86		-	-		-	-
-227		3	3.23		11	7.33		-	-		19	4.23		-	-
-229		-	-		6	4.00		7	3.38		15	3.34		3	3.09
202-211		8	8.60		10	6.67		-	-		23	5.12		3	3.09
204-211		3	3.23		-	-		-	-		-	-		-	-
206-219		2	3.92		-	-		-	-		-	-		-	-
210-219		32	34.41		42	28.00		67	32.37		141	31.40		39	40.21
-221		-	-		10	6.67		15	7.25		27	6.01		4	4.12
212-219		-	-		5	3.33		-	-		-	-		-	-
214-219		4	4.30		-	-		-	-		-	-		-	-
216-219		-	-		5	3.33		9	4.35		15	3.34		3	3.09

## References

[Abecasisetal2001] Abecasis G.R., Noguchi E., Heinzmann A., Traherne J.A., Bhattacharyya S., Leaves N.I., Anderson G.G., Zhang Y., Lench N.J., Carey A. (2001). Extent and distribution of linkage disequilibrium in three genomic regions. Am J Hum Genet.

[Angiusetal2002] Angius A., Bebbere D., Petretto E., Falchi M., Forabosco P., Maestrale B., Casu G., Persico I., Melis P.M., Pirastu M. (2002). Not all isolates are equal: Linkage disequilibrium analysis in Xq13.3 reveals different patterns in Sardinian sub populations. Hum Genet.

[Arnaiz-Villenaetal1995] Arnaiz-Villena A., Benmamar D., Alvarez M., Diaz-Campos N., Varela P., Gomez-Casado E., Martinez-Laso J. (1995). HLA allele and haplotype frequencies in Algerians. Relatedness to Spaniards and Basques. Hum Immunol.

[Arnaiz-Villenaetal1997] Arnaiz-Villena A., Martinez Laso J., Gomez Casado E., Diaz Campos N., Santos P., Martinho A., Breda Coimbra H. (1997). Relatedness among Basques, Portuguese, Spaniards, and Algerians studied by HLA allelic frequencies and haplotypes. Immunogenetics.

[Bellisetal2008] Bellis C., Cox H.C., Ovcaric M., Begley K.N., Lea R.A., Quinlan S., Burgner D., Heath S.C., Blangero J., Griffiths L.R. (2008). Linkage disequilibrium analysis in the genetically isolated Norfolk Island population. Heredity.

[Brancoetal2008a] Branco C.C., Cabrol E., São Bento M., Gomes C.T., Cabral R., Vicente A.M., Pacheco P.R., Mota Vieira L. (2008a). Evaluation of linkage disequilibrium on the Xq13.3 region: Comparison between the Azores Islands and mainland Portugal. Am J Hum Biol.

[Brancoetal2008b] Branco C.C., Pacheco P.R., Cabral R., Vicente A.M., Mota Vieira L. (2008b). Genetic signature of the São Miguel Island population (Azores) assessed by 21 microsatellite loci. Am J Hum Biol.

[Brancoetal2006] Branco C.C., Palla R., Lino S., Pacheco P.R., Cabral R., de Fez L., Peixoto B.R., Mota Vieira L. (2006). Assessment of the Azorean ancestry by Alu insertion polymorphisms. Am J Hum Biol.

[Brancoetal2008c] Branco C.C., São-Bento M., Gomes C.T., Cabral R., Pacheco P.R., Mota-Vieira L. (2008c). Azores Islands: Genetic origin, gene flow and diversity patterns. Ann Hum Biol.

[Bruges-Armasetal1999] Bruges-Armas J., Martinez-Laso J., Martins B., Allende L., Gomez-Casado E., Longas J., Varela P., Castro M.J., Arnaiz-Villena A. (1999). HLA in the Azores Archipelago: Possible presence of Mongoloid genes. Tissue Antigens.

[Guill1993] Guill J.H. (1993). A History of the Azores Islands.

[Helgasonetal2000] Helgason A., Siguroardóttir S., Nicholson J., Sykes B., Hill E.W., Bradley D.G., Bosnes V., Gulcher J.R., Ward R., Stefansson K. (2000). Estimating Scandinavian and Gaelic ancestry in the male settlers of Iceland. Am J Hum Genet.

[Laanetal2005] Laan M., Wiebe V., Khusnutdinova E., Remm M., Paabo S. (2005). X-Chromosome as a marker for population history: Linkage disequilibrium and haplotype study in Eurasian populations. Eur J Hum Genet.

[Latinietal2004] Latini V., Sole G., Doratiotto S., Poddie D., Memmi M., Varesi L., Vona G., Cao A., Ristaldi M.S. (2004). Genetic isolates in Corsica (France): Linkage disequilibrium extension analysis on the Xq13 region. Eur J Hum Genet.

[Martinez-Lasoetal1995] Martinez-Laso J., De Juan D., Martinez-Quiles N., Gomez-Casado E., Cuadrado E., Arnaiz-Villena A. (1995). The contribution of HLA-A, -B, -C and -DR, -DQ DNA typing to the study of the origins of Spaniards and Basques. Tissue Antigens.

[Meyeretal2006] Meyer D., Single R.M., Mack S.J., Erlich H.A., Thomson G. (2006). Signatures of demographic history and natural selection in the human major histo-compatibility complex loci. Genetics.

[Montieletal2005] Montiel R., Bettencourt C., Silva C., Santos C., Prata M.J., Lima M. (2005). Analysis of Y chromosome variability and its comparison with mtDNA variability reveals different demographic histories between islands in the Azores Archipelago (Portugal). Ann Hum Genet.

[Mota-Vieiraetal2005] Mota-Vieira L., Pacheco P.R., Almeida M.L., Cabral R., Carvalho J., Branco C.C., de Fez L., Peixoto B.R., Araújo A.L., Mendonça P. (2005). Human DNA bank in São Miguel island (Azores), a resource for genetic diversity studies. Progress in Forensic Genetics.

[Pachecoetal2005] Pacheco P.R., Branco C.C., Cabral R., Costa S., Araújo A.L., Peixoto B.R., Mendonça P., Mota Vieira L. (2005). The Y chromosomal heritage of the Azores Islands population. Ann Hum Genet.

[Reichetal2001] Reich D.E., Cargill M., Bolk S., Ireland J., Sabeti P.C., Richter D.J., Lavery T., Kouyoumjian R., Farhadian S.F., Ward R. (2001). Linkage disequilibrium in the human genome. Nature.

[Rosseretal2000] Rosser Z.H., Zerjal T., Hurles M.E., Adojaan M., Alavantic D., Amorim A., Amos W., Armenteros M., Arroyo E., Barbujani G. (2000). Y-chromosomal diversity in Europe is clinal and influenced primarily by geography, rather than by language. Am J Hum Genet.

[Santosetal2003] Santos C., Lima M., Montiel R., Angles N., Pires L., Abade A., Aluja M.P. (2003). Genetic structure and origin of peopling in the Azores islands (Portugal): The view from mtDNA. Ann Hum Genet.

[Schulzeetal2002] Schulze T.G., Chen Y.S., Akula N., Hennessy K., Badner J.A., McInnis M.G., DePaulo J.R., Schumacher J., Cichon S., Propping P. (2002). Can long-range micro-satellite data be used to predict short-range linkage disequilibrium?. Hum Mol Genet.

[Serviceetal2006] Service S., DeYoung J., Karayiorgou M., Roos J.L., Pretorious H., Bedoya G., Ospina J., Ruiz-Linares A., Macedo A., Palha J.A. (2006). Magnitude and distribution of linkage disequilibrium in population isolates and implications for genome wide association studies. Nat Genet.

[Shifmanetal2003] Shifman S., Kuypers J., Kokoris M., Yakir B., Darvasi A. (2003). Linkage disequilibrium patterns of the human genome across populations. Hum Mol Genet.

[Spinolaetal2005a] Spínola H., Brehm A., Bettencourt B., Middleton D., Bruges Armas J. (2005a). HLA class I and II polymorphisms in Azores show different settlements in Oriental and Central islands. Tissue Antigens.

[Spinolaetal2005b] Spínola H., Middleton D., Brehm A. (2005b). HLA genes in Portugal inferred from sequence-based typing: On the crossroad between Europe and Africa. Tissue Antigens.

[TheInternationalHapMapConsortium2007] The International HapMap Consortium (2007). A second generation human haplotype map of over 3.1 million SNPs. Nature.

